# Chronic Pemphigus (Pompholix Diutinus of Willan and Bateman)

**Published:** 1871-01

**Authors:** Walter Hay

**Affiliations:** Chicago


ARTICLE II. — *Chronic Pemphigus, (Pompholix diutinus of Willan and Bateman.)* By WALTER HAY, M.D., Chicago.

The subjoined analysis of a case of this disease is presented to the readers of the JOURNAL not only on account of its extreme rarity in this locality, but also, and more particularly, to illustrate the peculiar relations of the nervous system to cutaneous eruptions.

M. A. G——, a young girl aged about fifteen years; the second of six children, the four younger of whom have always enjoyed perfect health, born of healthy parents, both living, had suffered since early childhood, together with her older brother, from occasional attacks of cutaneous eruptions said to have resulted from the inoculation of spurious (?) vaccine, the same having been taken from a child having an eruption upon its scalp. The four younger children were not vaccinated from this source.

A long period of immunity from these attacks, characterized by the enjoyment of perfect health, was marked by no incident until the appearance of the catamenia in May last (1870) and their regular reappearance but once.

In August, (1870), her general health having been somewhat impaired since the interruption of the catamenia, bullæ appeared upon various portions of the cutaneous surface (face, trunk and extremities), and have continued to reappear up to the present time.

The bullæ are now apparent upon the cutaneous surface generally, but especially in the groins, upon the knees and in the popliteal spaces, where there are large patches denuded of epidermis and quite raw, preventing complete extension of the thighs and legs, embarrassing their movements, and causing flexion of the knees and hips, which, simulating contraction of the flexor tendons, interferes materially with locomotion.

There is great muscular debility, with some œdema about the face, neck and lower extremities, and pallor of the skin, lips and tongue, the latter having its surface smooth and its edges indented by the teeth. The pulse is small, sharp and wiry, though by no means accelerated, cardiac and respiratory functions feeble, but otherwise normal, alvine and renal evacuations said to be normal. Conjunctivæ pale, pupils widely dilated, responding to the influence of light regularly and uniformly but slowly. The urine (sanguinis) passed early in the morning contains much albumen. The total amount of urine secreted between 12 o'clock, noon, of one day until the same hour the next (the bladder having been previously emptied of its contents) was twenty-two and a half (22½) fluid ounces, having the specific gravity 1024, at the temperature of 60° (Fah.) and presented under the microscope (350 diameters) no deposit but epithelium and a few amorphous urates. The whole amount having been allowed to stand in a covered glass funnel, for fourteen hours, in a cool place, the specimen for examination being taken from the lower stratum in the neck of the funnel.

To analyze the case by a consideration of the morbid phenomena in the order of their objective prominence, we will take first the cutaneous eruption which presents all the evidences of destruction of epidermis with its exfoliation, occasioned by profuse serous effusion, resulting from acute inflammation, not confined to the vessels of the cutaneous glandular system, but including large tracts of the subcutaneous layer, involving the antecedent of obstructed circulation by capillary spasm. To corroborate this is the additional fact of the general pallor of surface, and next the condition of the pulse, as if to demonstrate the participation of the whole arterial system in this condition of spasm, increased arterial action, or, to go still farther back in the chain of physiological relations, exalted vaso-motor irritability.

Again, the dilatation of the pupil furnishes a reliable index of the degree (relative) of this irritability, and moreover assists in localizing it in the ganglionic nervous system. (*Pourfour du Petit*, *Arneman*, *Dupuy*, *Jno. Reid*, *Campbell*, *Excito Secretory System*, page 80.)

That the cerebro-spinal system is primarily exempted from the operation of the morbid influence, there is the negative evidence of freedom from perturbation of intellection, special sensation, general sensation and motion, (except as obstructed mechanically by the œdema), and by the continuity of the morbid conditions. All the evidence in the case thus far considered points in one direction, *i. e.*, to visceral congestion, and the next step must be the determination of the viscus or viscera involved primarily or secondarily, and from these to select the original offender. The comparative absence of physical signs of disease from most of the viscera, justifies their exclusion from any other than a secondary relation to the case, and the field of observation assuming more limited proportions, comprises only the urinary and reproductive organs. Of these, the first present an apparently wide departure from their normal status and functional energy, suggesting serious, indeed irreparable, structural lesion. The urine is highly albuminous, but at the same time accurate measurement demonstrates the fact that but twenty-two and a half ounces are secreted in twenty-four hours; now while the high specific gravity 1024, considered alone, would indicate an increase in the waste of tissue-forming solids of nearly fifty per cent., assuming Becquerel's standard of specific gravity of urine of women, 1015 to 1017, to be correct; yet the diminution in quantity, as compared with the standard of the same author (forty-seven ounces), being rather more than fifty per cent., the waste indicated by high specific gravity is corrected by diminished quantity. The aggregate waste, deduced according to Christison's formula, being 541·15 grains. The average excretion of solid matters by the kidneys in twenty-four hours is assumed by Golding Bird to be 650 grains, by comparison with which, the waste of solids by the kidneys in the case under consideration appears to be twenty per cent. less than it should be in health. Moreover, this waste consists principally of albumen, matter not yet having undergone oxydation. Now, a careful scrutiny of the sedimentary matters with the microscope fails to detect any tube casts or other renal debris, evidences of serious structural lesion, which, considered in connection with the last mentioned fact, not only forbids the localization of the primary lesion in the kidneys, but removes it to some point corresponding to a much earlier phase of the ascending metamorphosis of the blood.

Now, an essential lesion demonstrated in this investigation is diminished oxydation, and the analysis of the symptoms previously considered gave, as its result, spasm of the capillaries from exalted vaso-motor irritability, do not these two conditions stand correlated as cause and effect?

Exclusion has thus left but one organism to be examined, the reproductive system, and at this point, the collateral testimony, supplied in the history of the case may be applied, in which will be furnished the last link in the chain of evidence to establish the suspended catamenia as the ultimate efficient cause of the cutaneous eruption. Reasoning from analogy, and recognizing frequently the influence of a similar cause in the production of neuralgia or convulsion, and their opposite conditions, anæsthesia or paralysis, by reflex action through the spinal cord according as one or another portion of that center may be awakened to morbid activity or oppressed in functional energy by eccentric irritation of its appropriate conductors, the conclusion is logical that continued irritation of the vaso-motor fibres of the ganglionic system should induce modifications in the nutrition of distant parts by reflex action propagated through the ganglia of this system probably located in the posterior columns of the cord itself.

With regard to the probability of syphilitic infection, it must be acknowledged to rest upon very slender basis. In the first place, pemphigus presents none of the specific features of syphilitic eruption, its fancied resemblance to congenital syphilis being obscure; the coppery tint usually recognized as almost pathognomonic of the latter is altogether absent in this case. Secondly, its pathological phenomena occur in the inverse order as compared with those of syphilis, which, after the lapse of so long a time from the supposed infection, would have manifested themselves by much more essential modifications of plastic force, than in simple exfoliations of the cuticle, the cutaneous glandular apparatus constituting its first channel of exit from the economy. Thirdly, the relations of cause and effect between the inoculation of the virus and the eruption is not satisfactorily determined. For while it may be possible to inoculate the system with syphilis from a secondary pustule, it is utterly impossible to accomplish that result with vaccine. All so-called examples of spurious (?) vaccination should be subjected to much more rigid criticism than they have yet received. Fourthly, there is good ground for suspicion that the first appearance of the eruption was prior to the vaccination. All these data, in addition to the absence of syphilitic cachexia, are incompatible with the theory of syphilitic taint.

Moreover, the relation of pemphigus to syphilis has not been generally recognized by systematic writers. Wilson ignores it entirely, and suggests, as the cause of the eruption, intestinal (more especially uterine) irritation. Rayer, (quoted by the same author) attributes it, in one case, to suppressed hæmoptysis; and Duchesne Duparc (by the same), in a case of five years' duration associates it with arrested menstruation. Cazenave and Schedel, in their edition of 1847, coincide with Paul Dubois in regarding the pemphigus of newborn children as a "rare and serious form of *congenital syphilis:*" but in a subsequent edition (1852) speak of it as having "often been mistaken for a syphilitic affection." Nelligan is silent on this point, and vague and unsatisfactory generally. All the authorities, above referred to, agree in associating this malady in all its forms, whatever may be its exciting cause, with a debilitated state of the system at large, with poor and scanty food, over-exertion, low, damp dwellings, etc., as predisposing causes. And further, all of them indicate, vaguely and indefinitely, its *sympathetic* relations with various intestinal irritations, which they make no attempt to demonstrate or explain. By none is the relation of the nervous system to the disease even hinted at farther than may be comprehended under the word *sympathetic.*

